# Behavioral and cognitive factors influencing tick-borne disease risk in northeast China: Implications for prevention and control strategies

**DOI:** 10.1016/j.onehlt.2024.100736

**Published:** 2024-04-20

**Authors:** Ruying Fang, Sirui Li, Yunting Lyu, Xin Yang, Tingting Wang, Sen Li

**Affiliations:** a School of Environmental Science and Engineering, Huazhong University of Science and Technology, Wuhan 430074, PR China; b School of Art Design and Media, Wuhan Huaxia Institute of Technology, Wuhan 430223, PR China

**Keywords:** Tick-borne diseases, Preventive behaviors, Tick bites, Risk perception, Cognitive factors

## Abstract

The growth in ecotourism and nature-based recreational activities in China has resulted in an increased frequency of visits to green spaces, thereby elevating exposure to ticks and the subsequent risk of tick-borne diseases. This study comprehensively investigate individual behavioral and cognitive factors associated with the risk of contracting tick-borne diseases to facilitate the development of effective prevention and control strategies, supporting public health initiatives in high-prevalence regions. We conducted an extensive questionnaire survey among 3000 residents from three northeastern provinces in China (Heilongjiang, Jilin, and Liaoning), where tick-borne diseases exhibit relatively high prevalence. The survey focused on gathering information regarding participants' tick bite history, perception of tick-borne disease risks, and outdoor activity patterns. Using structural equations analysis, we explored the pathways and strengths of the associations between these factors. Our findings revealed an average self-reported tick bite rate of 14% among the participants. Notably, tick-borne encephalitis exhibited the highest self-reported prevalence of infection (4%) among tick-borne diseases, while both Lyme disease and Severe fever with thrombocytopenia syndrome had a prevalence of 2%. The average rate of tick bites among respondents' pets was 14%, with bites predominantly located on the ears, back, and abdomen. The strongest correlation was observed between tick bite rate and subsequent infections, emphasizing its role as the primary contributing factors to infectious status. Moreover, our results indicated that the causal structure of tick-borne disease infections varied across different cities, underscoring the significance of considering the ecological environment and regional knowledge on ticks. This study provides valuable insights into the current landscape of tick-borne disease infections in northeast China and identifies potential behavioral and cognitive factors, an aspect that has not been previously investigated. Our findings enable predictions on the future impact of knowledge dissemination efforts and improved urban facilities on mitigating tick bites and reducing tick-borne disease infections.

## Highlights

• Survey assessed tick exposure risk among 3000 residents and pets in northeastern China.
• Self-reported tick bites averaged 14%, with <5% prevalence of tick-borne disease infection.
• Residents with extensive tick knowledge had lower risk of contracting tick-borne diseases.
• No significant links found between preventive behaviors, occupations and tick-borne disease infections.
• Behavioral and cognitive factors' influence on infections varied by province.

## 1 Introduction

The main risk factors contributing to tick-borne diseases are associated with behaviors that increase the likelihood of exposure to tick bites [1]. Activities like outdoor sports, gardening, forestry, hunting, and logging are significantly linked to an elevated risk of contracting tick-borne diseases [2]. Since there is currently no vaccine available for most of tick-borne diseases, intervention strategies primarily focus on controlling and minimizing tick exposure to manage the associated risks [[3], [4], [5]].

Population exposure investigation focuses on understanding the transmission process of pathogens from vectors to vulnerable individuals. These studies investigates individual behaviors, patterns, and driving mechanisms that contribute to the risk of exposure in susceptible populations [6]. To quantify the risk of tick-borne diseases, it is crucial to comprehend not only the relative impact of individual variables but also the interactions between these influences. At the individual level, preventive measures against tick-borne diseases are multifaceted and include controlling the infection source, interrupting the transmission route, and protecting vulnerable populations [7]. Individual adoption of preventive behaviors can be influenced by various factors, such as socio-cultural and demographic characteristics, level of knowledge and perception of disease risk, and the prevailing disease incidence in a given area [8,9]. The main public health measures to prevent tick bites and tick-borne diseases include wearing protective clothing, using tick repellents on clothing and skin, showering or bathing after activities in high-risk areas, and regularly checking for ticks [10]. Surveys carried out in Poland, Slovakia [11] and Latvia [12] have shown that the level of public knowledge regarding preventing tick-borne diseases and understanding ticks still needs improvement.

Structural equation modeling (SEM) is a widely applied method for analyzing structural relationships that enables the testing of hypothetical causal dependencies between a range of variables [13]. It has been proposed that SEM allows for a more profound exploration of fundamental and theoretical issues compared to what traditional statistical methods can achieve [14]. SEM has been widely used in vectorial diseases, such as dengue [15] and schistosomiasis [16]. In the field of tick-borne disease investigation, SEM has been employed to estimate the risk of Lyme borreliosis post tick bites, taking into account factors such as tick developmental stage, tick engorgement, patient-estimated tick attachment duration, and patient age [17]. Furthermore, it has been utilized to explore the potential causal association between Bovine babesiosis infection and body weight fluctuations within cattle herds, thereby aiding farms in enhancing beef cattle productivity [18].

In China, after years of rewilding, there has been a surge in ecotourism and outdoor recreation. The frequency of people's visits to green spaces has risen sharply, leading to an increased risk of tick bites and subsequent transmission of tick-borne diseases. [19]. While understanding on the spatial distribution of vector ticks and pathogens has been well-established for China [[20], [21], [22]], there still exist significant gaps in understanding population exposure risk which may lead to pathogen spillover. Hence, it is crucial to comprehend the individual behavioral and environmental factors associated with risk and identify potentially effective prevention and control methods for tick-borne diseases, in order to support population health protection in areas with a high prevalence of such diseases.

In this study, we conducted an in-depth analysis of data collected from an extensive survey, focusing on the individual history of tick bites, risk perceptions of tick-borne diseases, and outdoor activity patterns among residents in three high-risk provinces for tick-borne diseases in China: Heilongjiang, Jilin, and Liaoning. Our main objectives were: (i) to investigate the rates of tick bites and occurrences of tick-borne disease infections in the northeastern region of China; (ii) to scrutinize the potential causal relationship among behavioral and cognitive factors, tick bites and instances of tick-borne disease infections. Additionally, we sought to explore regional variations to provide informed recommendations for formulating prevention and control policies that specifically target tick-borne diseases in high-risk areas.

## 2 Conceptual framework

A conceptual model was developed to examine the behavioral and cognitive factors influencing the causal relationship between tick-borne diseases and residents' behavior. The hypothetical paths associated with the factors in the conceptual model are represented using H0a-H5a, as illustrated in Fig. 2, Fig. 3. This model was subsequently tested using structural equation modeling to investigate its overall and comparative statistical significance over the three provinces.

Ticks can harbor a wide range of pathogens, encompassing bacteria like the spirochetes associated with Lyme disease, viruses such as the Powassan virus, and parasites like *Babesia*. In cases where ticks carry pathogens, these infectious agents can potentially infiltrate the host's system through the saliva, consequently triggering an infection (H0a). Previous studies have shown the risk of Lyme borreliosis reached 14.4% (95%CI 6.8%–24.6%) after one tick bite of a substantially engorged tick that tested positive for *Borrelia burgdorferi* s.l. DNA [17].

This study hypothesizes that there is a difference in occupational composition between urban and rural areas in China (H1a), with more people in the countryside working in agriculture and forestry-related jobs, resulting in greater exposure to tick habitats. A systematic evaluation and meta-analysis of factors influencing tick bites and tick-borne diseases has shown that environmental and behavioral factors significantly influence the risk of tick bites [23]. The familiarity with tick exposure among inhabitants of these regions may foster a heightened inclination toward comprehending ticks, recognizing the risks associated with disease transmission, and adopting preventative measures. Urban residents have greater access to information on tick-borne diseases, thereby enhancing their perception of associated risks (H1b). A study conducted in the northern part of Lublin province (eastern Poland) revealed that people living in rural areas had a significantly higher risk of tick bites [24]. However, the risk perception against tick bites remains low among residents in these areas. Urban areas, due to dense construction, and the comparably limited availability of natural open spaces, reduce tick habitats, whereas rural areas with a higher prevalence of forests, grasslands, and farmland raise tick encounter chances, heightening the risk of tick bites (H1c).

Investigations in Europe and the United States have highlighted the influence of perceptions of tick-borne disease and protective measures on the risk of tick bites in certain regions [25]. Those with greater awareness of tick-related risks tends to acquire knowledge about tick identification, adopt preventive measures against tick bites, and take precautions during outdoor activities. This study postulates that individuals with an elevated awareness of tick risks are more inclined to adopt preventive measures against tick bites. Individuals are more likely to avoid high-risk tick exposure activities and promptly seek appropriate treatment measures, this study hypothesizes that having a higher awareness of tick risks can reduce the risk of contracting tick-borne diseases. Even though surveys in Poland, Slovakia [11] and Latvia [12] have shown that the majority of people bitten by ticks, regardless of their medical background, do not follow appropriate medical treatment, we sought to investigate if this holds true in China. Understanding of tick presence, parasitic behavior, and the disease risks can stimulate people to prioritize tick concerns. The greater the extent of comprehension individuals possess concerning tick-borne diseases, the more inclined they become to implement precise preventive measures (H2b). Heightened awareness of tick-related risks facilitate accurate preventive measures, seeking suitable medical intervention following tick bites, and consequently diminishing associated risks (H2c).

The occupations of the respondents were categorized into farmers and non-farmers. It is hypothesized in this study that being a farmer influence tick awareness and outdoor activity patterns. Occupations associated with agriculture often involve extensive outdoor labor. Additionally, personal interests play a role in shaping the frequency of outdoor activities, such as leisure pursuits like hiking and camping (H3a). A study shows American farmers are at increased risk for tick bites, despite inadequate protective measures [26]. Farmers commonly operate within natural environments, potentially increasing their exposure to ticks and consequently deepening their understanding of tick-borne diseases risks [27]. Individuals in other professions can heighten their perception of risks posed by tick-borne diseases through the implementation of public health promotion and educational endeavors (H3b).

In highly endemic regions of Canada, spending more time in forests, woods, or tall grass increases the risk of tick bites [28]. Certain activities, such as yard work [29], outdoor work [30], and outdoor recreation like camping [31] and trail use [32] have been linked to increased disease infection. Ticks inhabit dense vegetation, including tall grasses, forests, and shrubs. Participation in outdoor pursuits such as camping and hiking could amplify individuals' potential for encountering ticks (H4a). If these ticks harbor disease-causing pathogens, there is a potential for the transmission of these pathogens into the host's body upon the bite, subsequently resulting in infection (H4b). This study assumes that individuals who prefer outdoor activities are more likely to take preventive measures against tick bites. Moreover, familiarity with tick presence and disease risks drives the adoption of protective actions during outdoor activities (H4c). The relationship between self-protection measures and disease outcomes has shown variation across studies. Some studies have found that bathing [33], using repellents [34], and performing tick checks [33,35] are associated with reduced disease, while others have not observed this association [29,31]. This study hypothesizes that preventive measures, including wearing garments with long sleeves and long pants, donning hats, and applying insect repellents, can effectively reduce tick contact with the skin (H5a).

## 3 Material and methods

### 3.1 Study area

**Fig. 1:**
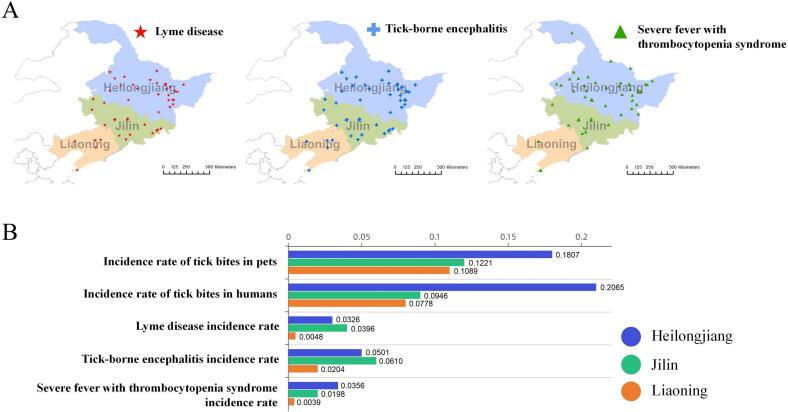
A) Self-reported of tick-borne disease incidences in the study area: Heilongjiang, Jilin and Liaoning. B) The tick bite rates and tick-borne disease infection rates in different provinces.

The ecological systems in the northeastern region of China exhibit complexity and diversity, characterized by abundant forested areas and a rich variety of wildlife and plant resources. This local natural conditions provide abundant habitat and host resources conducive to the survival and reproduction of ticks. For this study, questionnaires were distributed to 291 districts and counties in three north-eastern provinces in China. These provinces are acknowledged as high-risk regions for the occurrence of tick-borne diseases (e.g., Lyme disease (LD), tick-brone encephalitis (TBE), and severe fever with thrombocytopenia syndrome (SFTS)), including Heilongjiang, where the first incidence of Lyme disease was documented, along with two neighboring provinces, Jilin and Liaoning (Fig. 1A).

### 3.2 Data collection

In July 2022, an online survey was conducted to investigate tick risk perceptions, outdoor activity patterns, tick bite rates and incidences of disease infections among residents of Heilongjiang, Jilin and Liaoning provinces. The survey employed thoroughly designed questionnaires to evaluate the risk associated with behavioral exposure and its association with tick-borne diseases (Table S1). The survey was conducted in collaboration with Suzhou Zhongyan Network Technology Co., Ltd., a proficient service provider maintaining a substantial pool of around 45 million active users on Tabao.com, China's prominent online trading platform. This user database includes residents from almost every city across mainland China. Strict privacy protection regulations for personal information were implemented, along with incentives to encourage participation, thereby aiming to mitigate potential bias. A total of 3000 fully completed questionnaires were collected for this study, ensuring representation from a minimum of 10 responses per district or county.

### 3.3 Data analysis

The individual details related to occupations, preventive behaviors, lifestyles, risk perceptions, tick bites, and occurrences of tick-borne diseases, all of which were considered in the model (Table 1), were collected and processed. We utilized SEM to examine the hypothetical causal relationships proposed previously, interweaving multiple variables and assessing their direct and indirect impact on determining the specific outcome of interest, which, in this study, pertains to the incidence of tick-borne disease infection. The hypothesized causal relationships were visually depicted using a path diagram and subsequently subjected to examination for their standardized regression coefficients, thereby delineating the magnitude of direct causal influence. For this study, SEM was executed using IBM SPSS AMOS (version 23). The models were refined by adding or removing paths to ensure the Goodness-of-Fit (GOF) measures remained at adequate or recommended levels (Table S2). (See Table 2.)

**Table 1:** Behavior and cognitive factors analysis.

Symbols and the corresponding questions (sample size = 3000)		Mean	Standard deviation	Standardized regression weight
Preventive behavior (Rating from 0 to 4 for importance)				
PB1	Regularly inspecting my body for ticks and promptly removing them	2.43	0.017	0.917
PB2	Wearing long sleeves and trousers as protective attire.	2.82	0.016	0.0864
PB3	Fastening trousers at the ankles to mitigate exposure.	2.50	0.019	1.016
PB4	Employing insect repellent to deter tick attachment.	2.53	0.017	0.951
PB5	Exercising caution by avoiding proximity to tall trees and bushes.	2.62	0.016	0.901
Risk perception (Rating from 0 to 3 for importance)				
RP1	How concerned are you about the risk of tick bites when spending extended time in forested areas?	2.62	0.013	0.732
RP2	To what extent do you believe that tick distribution maps offer valuable insights to disease control departments?	2.58	0.014	0.742
RP3	In your opinion, can tick distribution maps play a role in enhancing public awareness about the risks associated with ticks?	2.67	0.013	0.695
RP4	To what degree are you surprised to learn that ticks are more widespread in our country than you previously thought?	2.54	0.014	0.753
RP5	How strongly do you agree that tick distribution maps should be accessible to a wider audience?	2.64	0.013	0.689
RP6	How necessary do you find having a detailed map illustrating the likelihood of tick distribution in your district and county of residence?	2.60	0.013	0.725
Bite risk				
BR1	Has your pet ever been bitten by a tick? (1 = Yes; 0 = No)	0.14	0.006	0.350
BR2	Have you or any member of your household experienced tick bites? (1 = Yes; 0 = No)	0.14	0.006	0.345
BR3	What is the combined count of tick bites on yourself, your family, and your pets in past two years?	0.40	0.018	0.959
Infection risk				
IR1	Have you, or any member of your household, ever suffered from Lyme disease? (1 = Yes; 0 = No)	0.02	0.003	0.155
IR2	Have you, or any member of your household, ever contracted tick-borne encephalitis? (1 = Yes; 0 = No)	0.04	0.004	0.201
IR3	Have you, or any member of your household, ever suffered from Severe fever with thrombocytopenia syndrome? (1 = Yes; 0 = No)	0.02	0.003	0.145
Location				
LC1	Is your place of residence in a rural area? (1 = Yes; 0 = No)	0.50	0.009	0.500
Occupation				
OP1	Are you involved in agriculture-related occupations? (1 = Yes; 0 = No)	0.06	0.004	0.240
Outdoor activity pattern				
OB1	Do you actively engage in outdoor activities? (1 = Yes; 0 = No)	0.71	0.008	0.455

**Table 2:** Socioeconomic characteristics of survey participants.

Socioeconomic	variables	Number of participants			Chi-square test	
		Heilongjiang	Jilin	Liaoning	Cramer's *V*	Probability
Gender	Male	722	332	574	0.039	0.102
	Female	595	323	454		
Age	≤18	0	0	0	0.044	0.296
	18–30	414	187	269		
	31–40	589	314	477		
	41–50	232	118	214		
	51–60	64	27	50		
	61–70	12	5	10		
	≥70	6	4	8		
Education	≤ Primary schools	20	22	27	0.120	0.000
	Junior middle school	112	62	186		
	Senior middle school	509	202	319		
	College	629	358	481		
	≥ Master	47	11	15		
Occupation	Governmental officer	38	20	80	0.155	0.000
	Specialist (Engineer, journalists, lawyer, professor, etc.)	103	41	47		
	Health care worker (Doctors, nurses, etc.)	80	12	26		
	Blue- or white-collar worker	505	250	383		
	Senior officials and business manager	95	33	25		
	Middle-level manager	102	77	54		
	Freelance worker	198	106	199		
	Student	96	44	99		
	Farmer	65	49	70		
	Housewife	11	10	16		
	Retiree	24	13	29		

## 4 Results

### 4.1 Participant demographics and characteristics

Among the 3000 participants included in the survey, there was slightly lager representation of males (1628) than females. The majority of participants fell within the age range of 18 to 50, a trend consistently observed across all three provinces. Regarding educational background, most respondents had completed at least a senior middle school education or higher. A specific subset of 184 individuals, accounting for 6% of the total sample, identified themselves as farmers in terms of occupation.

### 4.2 Self reported tick bites and disease infection patterns

A total of 607 respondents reported tick bites within the past 5 years, affecting themselves, family members, or pets, thus indicating a tick exposure rate of 20.23%. Among these occurences, 11 individuals reported being bitten by ticks on six occassions, while 155 people reported a single incidence. Biting incidents took place across diverse locations, encompassing forests, wetland, farmland, meadows, and house gardens. Approximately 42.27% of the respondents possessed the tools to remove the entire tick after a bite or chose to consult a nearby doctor. Interestingly, the incidence of tick bites appeared uniform between humans and pets, both reflecting a 14% occurrence rate. However, these rates varied substantially across provinces. In Heilongjiang, humans displayed a higher susceptibility to tick bites at 21%, compared to pets at 18% (Fig. 1B). Conversely, Jilin and Liaoning exhibited slightly higher rates of tick bites in pets as apposed to humans. The self-reported prevalence of TBE was found to be 4%, while the prevalence rates of SFTS and LD were 2%. Notably, the likelihood of contracting tick-borne diseases was higher in Heilongjiang and Jilin. Furthermore, respondents from Liaoning province demonstrated a tendency toward nearly negligible prevalence prevalence rates for SFTS and LD.

### 4.3 Possible interplay of factors influencing tick-borne disease infection risk

**Fig. 2:**
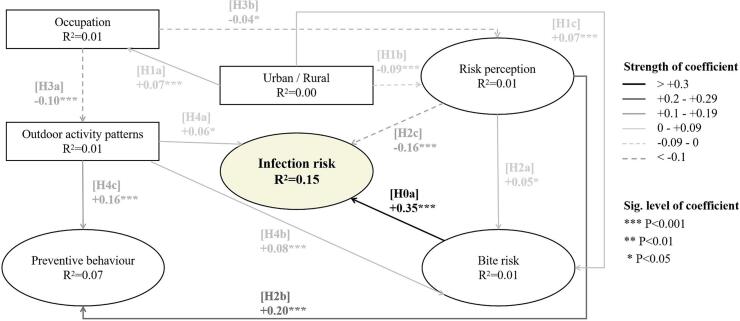
Causal relationships between behavior, cognitive factors, and tick-borne disease infection. Path coefficients are the magnitude or strength of relationships.

The association between tick bites and infection of tick-borne diseases exhibited substantial strength (coefficient = +0.35), making it the principal determinant of infectious status (Fig. 2). Moreover, heightened risk awareness significantly increased preventive behavior (coefficient = +0.20). Interestingly, although exposure to ticks increased among those more conscious of the associated risks (coefficient = +0.05), the likelihood of contracting tick-borne diseases displayed a tendency to decrease (coefficient = −0.16). Furthermore, individuals who engage in outdoor recreational activities were more likely to adopt protective measures against tick bites when venturing into the bush or forest regions (coefficient = +0.16). It is worth noting that there is an elevated risk of tick bites and tick-borne disease infections among people residing in in regions characterized by a higher prevalence of agricultural areas.

Fig. 2:Causal relationships between behavior, cognitive factors, and tick-borne disease infection. Path coefficients are the magnitude or strength of relationships.

### 4.4 The structural differences among the risk models in Northeast China's provinces

**Fig. 3:**
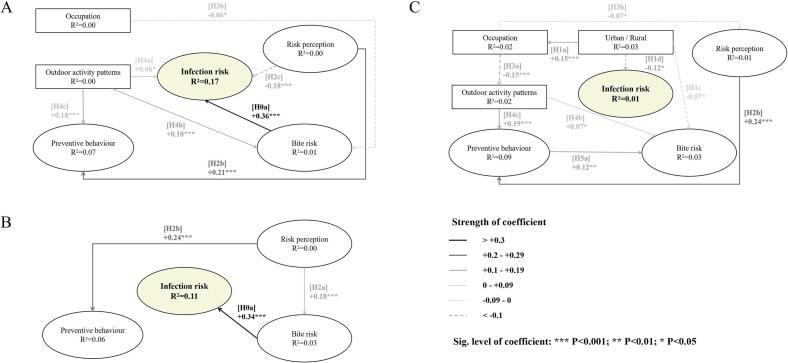
The differences in causal relationships between behavior, cognitive factors, and tick-borne disease infection among three provinces. A) Heilongjiang. B) Jilin. C) Liaoning.

The structural differences among the models for the three provinces in northeast China were evident (Fig. 3). There was a strong association between tick bites and infection risk, with coefficients of +0.36 and + 0.34 for Heilongjiang and Jilin, respectively. However, this path was not significant in Liaoning province, resulting in an R^2^ value of only 0.01. The highest R^2^ value for the risk of tick-borne disease infection was 0.16 in the Heilongjiang model. Notably, only in Liaoning province did a significant association emerge between participants' place of residence and their occupations. Those residing in rural areas had an increased probability of identifying as farmers (coefficient = +0.15), while farmers had a weaker perception of the risk of tick-borne disease (coefficient = −0.07). In contrast, Jilin province featured the fewest hypothetical pathways, with only three final pathways retained. None of the environmental factors in the Jilin model held validity, underscoring the pivotal role of individual risk awareness and preventive behavior. Among these factors, respondents' heightened risk awareness positively influenced their adoption of preventive measures against ticks (coefficient = +0.24), and those who were more aware of the risks also exhibited greater propensity for tick exposure (coefficient = +0.18).

Fig. 3:The differences in causal relationships between behavior, cognitive factors, and tick-borne disease infection among three provinces. A) Heilongjiang. B) Jilin. C) Liaoning.

## 5 Discussion

### 5.1 Tick-borne disease risk patterns in northeastern China

Across the three provinces of northeastern China, the mean rate of self-reported tick bites averaged 14%, and instances of tick-borne disease infection were observed in <5% of cases. The model's interpretability exhibited a higher degree of coherence in the overall participant sample as well as in Heilongjiang and Jilin provinces, yet presented a relatively lower coherence in Liaoning province. Notably, the tick bite rate observed in Niigata Prefecture, Japan, during the survey was 4.8% [36], which is notably similar to the findings of this study. However, a study in Sweden showed that 31% of the respondents report one or more tick bites in the last year [37].

### 5.2 Dominance of individual behavioral factors

An important revelation from this study is the prominence of individual behavioral determinants in influencing both the risk of exposure to and the subsequent infection of tick-borne diseases. In contrast, the influence of environmental factors demonstrated a relatively modest effect. This underscores the pivotal role of personal actions and choices in shaping the incidence of tick-borne diseases within the populace.

Our results suggests that a preference for outdoor activities exhibited a positive effect (coefficient = +0.09) on tick exposure, leading to an increased risk of tick bites, particularly in forests and grasslands. Interestingly, heightened awareness of the tick-borne disease risks corresponded with an elevated possibility to tick bite. This could be attributed to individuals' past experiences with tick bites, which heightened their concern and understanding about ticks and related diseases and subsequent inclination to acquire knowledge on preventing future tick bites. For instance, participants with better understanding of tick-borne diseases demonstrated a higher tendency to undertake preventive measures such as wearing long sleeves and trousers or using insect repellents while traversing vegetated landscapes. This finding is in line with the investigation in Long Island, New York, USA, which pointed that self-reported concern on tick-borne diseases was significantly and positively associated with tick bite prevention practices [38]. Moreover, a majority of respondents (74.33%) expressed a strong desire to acquire further knowledge regarding tick-borne diseases. Research conducted in Sweden has shown that one way of increasing the use of protective measures could be to actively inform people of the effectiveness of the different measures [37]. Therefore, it is imperative for local governments facing a high prevalence of tick-borne diseases to intensify their efforts to their public education initiatives concerning ticks.

Residing in areas with substantial farmland proportions elevates the risk of tick exposure. While environmental factors play a secondary role in comparison to other determinants, they should not be overlooked in the transmission of tick-borne diseases among populations. With the increasing public interest in outdoor activities in the study area, urban area host a large number of outdoor enthusiasts, whose visits to forests and woodlands for camping, bicycling, and hiking [39] could increase their exposure and subsequent infection risks. The presence of woodland, stone walls, and accumulated leaf litter in yards is associated with an increased incidence of tick-borne diseases [35]. In response, public health officials should have recommended property management strategies, including leaf litter removal, brush clearing, and installation of barriers between lawns and wooded areas, aimed at reducing ticks and averting tick bites.

### 5.3 Region-specific variation in causal models

The structural composition of the causal model, encompassing paths and coefficients, exhibited significant diversity among the three provinces under examination. This disparity implies the likelihood of distinctive region-specific variables that exert a substantial impact on individual susceptibility to tick-borne diseases. This underscores the necessity of considering local nuances while devising targeted strategies for the mitigation and management of tick-borne diseases.

Environmental factors inherent to different regions can contribute to variations in tick distribution. Heilongjiang, characterized by extensive forest cover, particularly boreal coniferous forests that provide an ideal habitat for ticks [40]. In contrast, Jilin exhibits a relatively diverse range of vegetation, encompassing forests, grasslands, and cultivated land. Unlike the other two provinces, Liaoning, being coastal, displays a lesser extent of natural vegetation cover. The results of the questionnaire survey indicated varying tick bite rates across the three provinces, with the highest rate observed in Heilongjiang, followed by Jilin, and then Liaoning (Fig. 1B). Moreover, respondents in Liaoning exhibited comparatively lower levels of Preventive behavior compared to the other provinces. In the Heilongjiang model, the factors associated with tick-borne disease infection were outdoor activity patterns, risk perception, and bite risk. In the Jilin and Liaoning models, there was only one pathway associated with tick-borne disease infection, which was bite risk and residential area (Urban/Rural) respectively. The Heilongjiang and Liaoning models retain pathways linked to respondents' occupation, place of residence, and outdoor activity patterns, while these factors are excluded in the Jilin model. The economy of Heilongjiang province is dominated by agriculture and heavy industry, potentially leading to pronounced economic disparities between urban and rural areas. In Liaoning, the economy demonstrates diversity, spanning industry, agriculture, and services, with coastal port trade playing a significant role. Among the three provinces, respondents in Liaoning exhibited the highest level of risk awareness and demonstrated a greater inclination to acquire knowledge about ticks from tick distribution maps (Fig. 1C). Meanwhile, Jilin Province hinges its economy largely on industry, with sectors like automobile manufacturing holding substantial prominence. Regarding outdoor activity pattern, approximately one-third of respondents in Liaoning and Heilongjiang actively engage in outdoor activities, while in Jilin, this proportion decreased to one-fifth.

### 5.4 Limitations and future directions

Notably, due to limited prior surveys on risk perceptions and protective behaviors related to tick-borne diseases in China, evaluating potential sample bias and its extent is challenging. For example, the survey results indicate instances where 31 respondents had contracted tick-borne diseases but had not indicated prior tick bites. The potential reasons for this phenomenon include the incubation period of these diseases, which might lead to forgetting or not recognizing a tick bite. This inaccuracy may lead an impact on the results of the association between tick bites and tick-borne disease infections in the model.

Globally, climatic patterns may determine the broad distribution of ecological hazards and exposure risks. To quantify the risk of tick-borne diseases, it is crucial to comprehend not only the relative impact of individual variables but also the interactions between these ecological and non-ecological influences. These complex interactions have a profound impact on the intricate ecological relationship among vectors, pathogens, and hosts, serving as the primary driving forces behind the spread of tick-borne diseases, acting a direction worth delving into for the future.

## 6 Conclusions

This investigation sheds light on the prevalence of tick bites and tick-borne disease infections in northeastern China. It underscores the pivotal role of individual behaviors in influencing exposure and infection rates while highlighting the complex interplay of factors that varies among different provinces. From a One Health perspective, controlling tick-borne zoonotic diseases requires understanding socio-ecological factors of exposure risk, and devising collaborative strategies that includes wildlife management, habitat conservation, and public education to reduce tick bites on both residents and their pets. Such insights are indispensable for devising targeted strategies to mitigate the impact of tick-borne diseases and enhance public health outcomes.

## Declaration of competing interest

The authors declare that they have no known competing financial interests or personal relationships that could have appeared to influence the work reported in this paper.

## Data Availability

Data will be made available on request.

## Appendix A Supplementary data

Suppl. Table 1. Summary of questions used in survey and related variables. Suppl. Table 2. Goodness-of-fit measures (GOFs) for the confirmatory factor analysis and structural equation models. Image 1
