# Supplementary material for: Behavioral and cognitive factors influencing tick-borne disease risk in northeast China: Implications for prevention and control strategies
Source: One Health. 2024 Apr 20;18:100736. doi: 10.1016/j.onehlt.2024.100736 (PMC11061341; doi:10.1016/j.onehlt.2024.100736)
Supplement: Supplementary file 1 — Suppl. Table 1. Summary of questions used in survey and related variables. Suppl. Table 2. Goodness-of-fit measures (GOFs) for the confirmatory factor analysis and structural equation models. [file mmc1.docx]

Table S1 Summary of questions used in survey and related variables

| **Questions and related variables** | | **Type** | **Definition/Options** | | |
| --- | --- | --- | --- | --- | --- |
| **Preventive behaviour** | | | | |  |
|  | Regularly inspecting my body for ticks and promptly removing them | Ordinal | 1=No knowledge at all；2=Just heard of it；3=Some knowledge；4=Very familiar | | |
|  | Wearing long sleeves and trousers as protective attire. | Ordinal |  |  |  |
|  | Fastening trousers at the ankles to mitigate exposure. | Ordinal |  |  |  |
|  | Employing insect repellent to deter tick attachment. | Ordinal |  |  |  |
|  | Exercising caution by avoiding proximity to tall trees and bushes. | Ordinal |  |  |  |
| **Risk perception** | |  |  | | |
|  | How concerned are you about the risk of tick bites when spending extended time in forested areas? | Nominal | 1=I disagree；2=I don't know；3=I agree | | |
|  | To what extent do you believe that tick distribution maps offer valuable insights to disease control departments? | Nominal |  |  |  |
|  | In your opinion, can tick distribution maps play a role in enhancing public awareness about the risks associated with ticks? | Nominal |  |  |  |
|  | To what degree are you surprised to learn that ticks are more widespread in our country than you previously thought? | Nominal |  |  |  |
|  | How strongly do you agree that tick distribution maps should be accessible to a wider audience? | Nominal |  |  |  |
|  | How necessary do you find having a detailed map illustrating the likelihood of tick distribution in your district and county of residence? | Nominal |  |  |  |
| **Bite risk** | |  |  | | |
|  | Has your pet been bitten by a tick? | Nominal | 0=False 1=True | | |
|  | Have you or any member of your household experienced tick bites? | Nominal |  |  |  |
|  | What is the combined count of tick bites on yourself, your family, and your pets in past two years? | Continuous |  | | |
| **Infection risk** | |  |  | | |
|  | Have you, or any member of your household, ever suffered from Lyme disease? | Nominal | 0=False 1=True | | |
|  | Have you, or any member of your household, ever contracted tick-borne encephalitis? | Nominal |  |  |  |
|  | Have you, or any member of your household, ever suffered from Severe fever with thrombocytopenia syndrome? | Nominal |  |  |  |
| **Location** | |  |  | | |
|  | Is your place of residence in a rural area? | Nominal | 0=False 1=True | | |
| **Outdoor activity pattern** | |  |  | | |
|  | Do you actively engage in outdoor activities? | Nominal | 0=False 1=True | | |
| **Which categories best describe you?** | |  | |  |  |
|  | Gender | Nominal | 0 = Male;1= Female | | |
|  | Age | Ordinal | 1 = 0~18; 2 = 18~30; 3 = 31~40; 4 = 41~50; 5 = 51~60, 6 = 61~70, 7 = 70+ | | |
|  | Occupation | Nominal | 1 = Governmental officer; 2 = Specialist (Engineer, journalists, lawyer, professor, etc.); 3 = Health care worker (Doctors, nurses, etc.); 4 = Blue- or white-collar worker; 5 = Senior officials and business manager; 6 = Middle-level manager; 7 = Freelance worker; 8 = Student; 9 = Farmer；10=Housewife；11=Retiree | | |
|  | Education | Nominal | 1=Primary school and below; 2 = Junior middle school; 3 = Senior middle school; 4= College; 5 = Master’s degree or above | | |

Table S2 Goodness-of-fit measures (GOFs) for the confirmatory factor analysis and structural equation models

| GOFs * | Adequate level | Recommended level | Total | Heilongjiang | Jilin | Liaoning |
| --- | --- | --- | --- | --- | --- | --- |
| Root mean sq. error of approx. (RMSEA) | < 0.1 | < 0.05 | 0.032 | 0.035 | 0.033 | 0.035 |
| Standardised root mean sq. residual (SRMR) | < 0.1 | < 0.05 | 0.034 | 0.042 | 0.041 | 0.043 |
| Goodness-of-fit index (GFI) | > 0.9 | > 0.95 | 0.979 | 0.971 | 0.966 | 0.965 |
| Adj. goodness-of-fit index (AGFI) | > 0.8 | > 0.9 | 0.972 | 0.962 | 0.954 | 0.956 |
| Comparative fit index (CFI) | > 0.9 | > 0.95 | 0.951 | 0.944 | 0.966 | 0.946 |
| Tucker–Lewis index (TLI) | > 0.9 | > 0.95 | 0.943 | 0.934 | 0.959 | 0.937 |
| Normal fit index (NFI) | > 0.9 | > 0.95 | 0.936 | 0.913 | 0.923 | 0.907 |
| Incremental fit index (IFI) | > 0.9 | > 0.95 | 0.951 | 0.945 | 0.966 | 0.946 |
| Relative fit index (RFI) | > 0.9 | > 0.95 | 0.925 | 0.897 | 0.907 | 0.892 |

*χ² test statistics (χ² probability level and χ²/degrees of freedom) are also frequently used GOFs. However, they are recommended not to use when the sample size is large*. (Jöreskog, Karl G., and Dag Sörbom. LISREL 8: Structural equation modeling with the SIMPLIS command language. Scientific Software International, 1993.)*

The GOFs also used for confirming the predictive validity and reliability of the constructs in the confirmatory factor analysis
